# Osmotic-shock produced by vitrification solutions improves immature human oocytes in vitro maturation

**DOI:** 10.1186/s12958-016-0161-1

**Published:** 2016-05-11

**Authors:** Inmaculada Molina, Judith Gómez, Sebastián Balasch, Nuria Pellicer, Edurne Novella-Maestre

**Affiliations:** Unidad de Reproducción Humana, Área de Salud de la Mujer, Hospital Universitario y Politécnico La Fe, Valencia, Spain; Departamento de Estadística e Investigación Operativa Aplicadas y Calidad, Universidad Politécnica de Valencia, Valencia, Spain; Fundación Instituto Valenciano de Infertilidad, Instituto Universitario IVI, Valencia, Spain; Unidad de Genética, Torre A planta 4º, Hospital Universitario y Politécnico La Fe, Avenida de Fernando Abril Martorell, nº 106, 46026 Valencia, Spain; Grupo de investigación de Medicina Reproductiva, Instituto de Investigación Sanitario La Fe, Valencia, Spain

**Keywords:** In vitro maturation, Vitrification, Immature oocytes, Fertility preservation, Contingency tables

## Abstract

**Background:**

During cytoplasmic oocyte maturation, Ca^2+^ currents are vital for regulating a broad range of physiological processes. Recent studies have demonstrated that DMSO and EG cause large transient increases in intracellular Ca^2+^ in mouse oocytes. The CP used in vitrifying protocols also increases the intracellular calcium transient. The aim of this study is to evaluate the effects of vitrifying time (before and after IVM) and exposure to the vitrification solutions and ionomycin on oocyte quality and embryonic development.

**Methods:**

221 GV-oocytes unsuitable for IVF-ICSI cycles were randomly distributed into one of the following three groups. G1 (control group): 41 GV-oocytes IVM until MII; G2: 43 oocytes vitrified at GV stage and IVM until MII stage; and G3: 53 GV-oocytes IVM until MII and then vitrified. In order to clarify the effect of vitrification solutions (VS) on human oocyte IVM through the intracellular Ca^2+^ oscillation, the following two groups were also included. G4: 43 GV-oocytes exposed to VS and IVM until MII; and G5: 41 GV-oocytes exposed to ionomycin and IVM until MII. All GV-oocytes that reached MII-stage were parthenogenetically activated to assess oocyte viability. IVM was performed in IVF-medium (24–48 h). Chemical treatment (ionomycin) and osmotic treatment (vitrification solutions) were performed without liquid-N_2_ immersion. The following rates were evaluated: survival (SR), in-vitro maturation (IVMR), activation (AR), development to 2-cell (DRC), development to morula (DRCM) and development to blastocyst (DRB). Ratios between the different treatment groups were compared using contingency tables analysis (chi-square test).

**Results:**

A high survival rate was obtained in G2 (95.5 %) and G4 (96.6 %). In-vitro maturation rate was significantly higher for G4 (86 %) and G2 (83.7 %) compared to G1 (63.4 %), G3 (56.6 %) and G5 (48.8 %). DRCM was significantly higher for G1 and G2 compared to G3 (G1: 15.8 %, G2: 20.7 % and G3: 0 %). DRB was only obtained for the oocytes vitrified before IVM (G2: 3.4 %). AR was also significantly higher for G2 and G4 compared to G5 (G2: 80.5 %, G4: 86.5 % and G5: 55 %). DRCM and DRB were only obtained in G2 and G4. DRCM was significantly higher for oocytes vitrified at GV stage (G2) and for oocytes exposed to the VS in G4 compared to the oocytes exposed to the ionomycin in G5 (G2: 20.7 %; G4: 37.5 % and G5: 0 %).

**Conclusions:**

Vitrifying GV-oocytes improves their IVM. Further investigation could look to increase the oocyte pool and improve fertility preservation options.

## Background

Recent advances in the development of protocols for the cryopreservation of germ cells have resulted in the introduction of vitrification as a routine clinical technique for oocyte cryopreservation. Using vitrification, in contrast to the conventional slow-freezing method, has improved the efficacy of human oocyte cryopreservation and pregnancy outcomes [1–12], and mature oocyte vitrification has thus become an important tool for women who are at risk of losing ovarian function from radiation, chemotherapy and surgery.

One of the advantages of vitrification techniques is their application in the field of fertility preservation due to delayed motherhood or for oncological reasons. The problem that we often find in the clinical practice of these patients is a poor ovarian response to stimulation. These patients therefore have to undergo several ovarian stimulations to obtain an adequate pool of mature oocytes to ensure a future pregnancy. For some oncological patients, ovarian stimulation to obtain mature oocytes is contraindicated due to the disease itself or the age of the patient.

For these special cases, immature oocyte retrieval followed by in vitro maturation (IVM) and cryopreservation of oocytes or embryos can be considered [13]. It has been shown, however, that the implantation and developmental potential of embryos derived from IVM oocytes is significantly lower than in vivo matured oocytes. Therefore, IVM methodology has remained largely empirical and, in consequence, relatively inefficient [14].

Oocyte maturation involves nuclear maturation and cytoplasmic-coordinated modifications [15] necessary for fertilization and early embryonic development. However, one of the main problems of oocyte IVM is the asynchronicity between nuclear maturation and cytoplasmic modifications. Nuclear maturation after an oocyte IVM process is easy to detect because it involves GV breakdown (GVBD) and the extrusion of the first polar body (PB). However, the functional changes that take place during the cytoplasmic maturation are difficult to evaluate during the clinical practice without damaging the oocyte. It is known that during cytoplasmic oocyte maturation, Ca^2+^ currents are vital for regulating a broad range of physiological processes [16, 17]. Ca^2+^ chelators block GVBD in mammalian oocytes at least up to the first metaphase [18]. In absence of intracellular Ca^2+^ elevation, spontaneous meiosis resumption in vitro does not occur [19]. Furthermore, it has been demonstrated that the injection of Ca^2+^ in mouse oocytes induces parthenogenetic activation and subsequent normal embryo development [20].

It has been shown that some cryoprotectants, such as dimethyl sulfoxide (DMSO) and ethylene glycol (EG), cause a transient intracellular Ca^2+^ rise in various cell lines [21, 22], and that the fusion of cortical granules to the oocyte plasma membrane is also Ca^2+^-dependent [23, 24]. This suggests that these cryoprotectants trigger cortical granule release by increasing intracellular Ca^2+^.

Recent studies have demonstrated that DMSO and EG cause large transient increases in intracellular Ca^2+^ in mouse oocytes, which induces zona hardening and significantly reduces fertilization [25, 26].

These data led us to the assumption that increasing intracellular Ca^2+^ transient produced by these cryoprotectants in vitrification protocols might help immature oocytes, as increasing intracellular Ca^2+^ transient is capable of contracting the endoplasmic reticulum (ER). This leads to the release of Ca^2+^, which is essential for GVBD and meiosis resumption.

In clinical practice, vitrification of mature oocytes, and in some cases the IVM of GV oocytes for subsequent vitrification and storage if they reach the stage of M2, is a routinely established technique. To date, however, IVM protocols have given poor efficiency results.

These data led us to think that the vitrification process helps the immature oocyte in the maturation process by regulating the synchronicity between nuclear maturation and cytoplasmic modifications. Therefore, for those patients needing to preserve their fertility for different reasons, immature oocytes that are discarded after the ovarian puncture can be vitrified promoting maturation process after devitrification for in order to obtain optimal mature gametes for fertilization.

The main objective of this study was to evaluate the effects of vitrification solutions (VS) on human oocyte IVM.

## Methods

This study was approved by the Institutional Review Board of the Hospital Universitario y Politécnico La Fe de Valencia in Spain. Patients included in the study were fully informed and gave their signed consent. All procedures for consent, method and sections were compliant with the ethical guidelines approved by the Ethics Committee.

### Patients and GV-oocytes

A total of 221 GV-oocytes from 112 ICSI cycles performed between November 2013 and August 2014 were used in this study. These oocytes were not suitable for an ICSI cycle, however they are an ethical and legal biological material that can be used to improve cryopreservation and IVM protocols for further clinical application in humans.

All patients were less than 36 years old. Cycles with endometriosis, low response, and ovarian failure were excluded.

The stimulation protocol has been described before [27]. After ovum pick-up, oocyte-cumulus complexes were denuded in order to assess nuclear maturity. The cumulus-oocyte complexes (COCs) with immature appearance were partially denuded until the GV was observed. All partially denuded oocytes with a GV structure and no polar body in the perivitelline space were considered immature and included in this study. Only morphologically normal oocytes were included.

### Experimental design

In order to evaluate the effects of cryopreservation on GV human oocytes vitrified before and after IVM, 221 oocytes were distributed into the following groups:

Group 1 (G1: Control group): 41 GV-oocytes in vitro matured until MII; Group 2 (G2): 43 GV-oocytes vitrified at GV stage, warmed, and in vitro matured until MII; and Group 3 (G3): 53 GV-oocytes in vitro matured until MII and then vitrified.

**Fig. 1:**
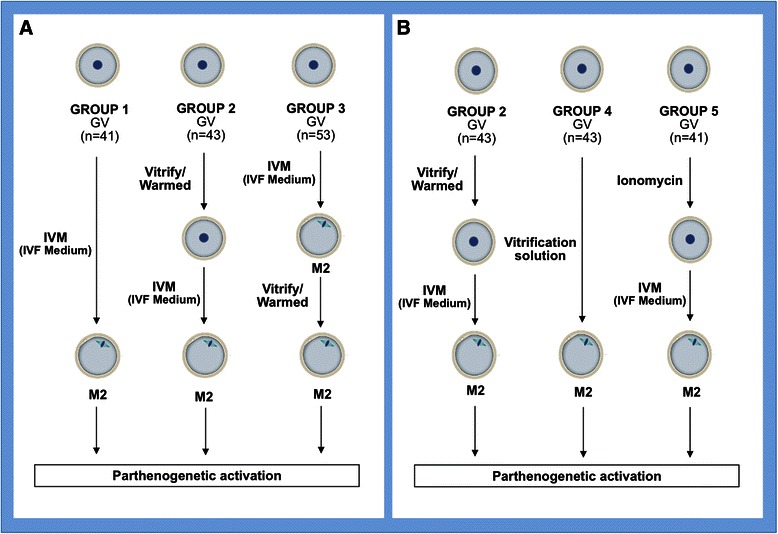
Experimental design. The experimental design included the following experimental groups: **a** Effects of cryopreservation on GV human oocytes vitrified before and after IVM. Group 1 (G1, Control group): 41 GV-oocytes in vitro matured until MII. Group 2 (G2): 43 GV-oocytes vitrified at GV stage warmed and in vitro matured until MII. Group 3 (G3): 53 GV-oocytes in vitro matured until MII and then vitrified. **b** Effects of vitrifying solutions (VS) on human oocyte IVM through the intracellular Ca^2+^ oscillation. In this case, G2 (oocytes vitrified at GV stage) was used as control group. Group 4 (G4): 43 GV-oocytes exposed to vitrification solutions (equilibration solution and vitrification solution from Vitrification Kit media, Irvine®, Denmark) without the subsequent immersion in liquid nitrogen (LN_2_) and in vitro matured until MII. Group 5 (G5): 41 GV-oocytes exposed to 10 μM ionomycin and in vitro matured until MII. All the MII oocytes obtained were cultured in vitro for 2 h and then parthenogenetically activated in order to assess the oocyte cytoplasmic maturity and viability

In order to evaluate the effect of vitrification solutions (VS) on human oocyte IVM through the intracellular Ca^2+^ oscillation, GV-oocytes were also exposed to different substances that interact with the Ca^2+^ pattern, such as VS (osmotic activation) and ionomycin (chemical activation). In this case, G2 (oocytes vitrified at GV stage) was used as a control group. The groups were as follows: Group 4 (G4): 43 GV-oocytes exposed to vitrification solutions (equilibration solution and vitrification solution from Vitrification Kit media, Irvine®, Denmark) without the subsequent immersion in liquid nitrogen (LN_2_) and in vitro matured until MII; and Group 5 (G5): 41 GV-oocytes exposed to 10 μM ionomycin and in vitro matured until MII (see activation protocol by Paffoni et al. [28]). The experimental design scheme is shown in Fig. 1.

In order to avoid variations due to culture conditions, all oocytes were cultured in the same incubator. Moreover, oocytes from G1 and G3 were simultaneously cultured and oocytes from G2 and G3 were simultaneously devitrified. Oocytes from G4 and G5 were also simultaneously treated and cultured. Only the oocytes that survived the vitrifying process were included in the study.

All MII oocytes obtained were cultured in vitro for 2 h and then parthenogenetically activated in order to assess the oocyte cytoplasmic maturity and viability.

### Oocyte In vitro maturation

The GV-oocytes were matured in vitro via culturing in 10 μL of IVF (ORIGIO MediCult, Denmark) micro-drops covered with mineral oil. Oocytes which expelled the first polar body between 24 and 48 h of in vitro culture were considered MII mature oocytes. The nuclear maturity was assessed at 24 and 48 h after in vitro culture. The IVM rate (IVMR) was calculated as the percentage of GV-oocytes reaching the MII stage between 24 and 48 h after in vitro culture (IVMR = number of MII oocytes/total number of GV-oocytes × 100).

### Vitrifying and warming oocytes

Oocytes were vitrified following a standard protocol (Vitrification Kit media, Irvine, Denmark) using aseptic devices (CryoBiosystem, VHS Kit, CryoTip ® Loading Protocol Irvine). Oocytes were gradually exposed to the equilibration solutions (ES) as follows: one minute in ES-1, two minutes in ES-2, and 6–10 min in ES-3. Afterwards, they were transferred from ES-3 to the vitrification solution (VS) for 30 s before loading. Then, the oocytes were loaded in CryoTip devices and immersed into the LN_2_ for 80 s.

To warm the oocytes, the CryoTip was taken out of the LN_2_ and within 1 s fully immersed in a 37 ° C water bath and gently agitated for 3 s. The oocytes were rinsed in the dilution solution (DS) for 4 min and transferred to the washing solutions (WS-1 and WS-2) for 4 min each. The warmed oocytes were then transfered to an incubated IVF culture medium with 20 % (v/v) protein supplement for 2 h. Oocytes that showed clear bright homogeneous cytoplasm and an intact plasma membrane and zona pellucida were considered to have survived the vitrifying process. The survival rate (SR = number of survived oocytes/total number of vitrified oocytes × 100) after warming was only calculated for G2 (oocytes vitrified at GV) and G3 (oocytes vitrified at MII).

### Parthenogenetic oocyte activation

To assess oocyte viability after warming, the chemical parthenogenetic activation protocol described in Paffoni et al. [28] was performed with minor modifications. MII were exposed to a concentration of 10 μM of ionomycin in IVF medium for 5 min in the dark. They were then washed twice and incubated for 3 h in 2 mM of 6-dimethylaminopurine (6-DMAP) (Sigma-Aldrich Srl, Milan, Italy) in ISM1 culture medium (ORIGIO MediCult). Subsequently, oocytes were washed three times in the same medium and cultured for 18–20 h more until the chemical activation was assessed. Those eggs that showed an elongated pronucleus and had not expelled the second polar body were considered activated.

### Assessment of parthenogenetic embryonic development

The success of the parthenogenetic chemical activation for inducing cleavage and embryonic development, as well as the developmental stages of embryos (ranging from 2-cell to blastocyst), were evaluated. Parthenogenetic embryos were washed twice and cultured in ISM1 medium for 3 days and ISM2 medium (ORIGIO MediCult) until day 5 (120 h after activation) when the blastocyst formation was assessed.

Activation rate (AR) was calculated as the number of MII that showed signs of activation out of the total number of MII cultured in 10 μM of ionomycin. The development rate to cleavage or to 2-cell embryos (DRC), the development rate to compacted morula (DRCM), and the development rate to blastocyst (DRB) were calculated, respectively, as a ratio of the total number of embryos that developed to 2-cells on day 2, compacted morula on day 4, and blastocyst on day 5, to the total number of activated oocytes. Blastocysts were classified using the system of Gardner and Schoolcraft [29].

In order to clarify the results, the development rate was calculated using the number of activated oocytes for all the stages evaluated (DRC = number of 2-cells embryos/total number of activated oocytes × 100; DRCM = number of morula/total number of activated oocytes × 100; DRB = number of blastocysts/total number of activated oocytes × 100).

### Statistical analysis

Ratios between different treatment groups were compared using contingency tables analyses with Statgraphics Centurion XVI.II.

A *p*-value ≤ 0.05 in the corresponding chi-square test was considered statistically significant.

## Results

### Effects of vitrifying before and after IVM on oocyte viability, IVM and embryo development

The survival rate (SR) after warming was only evaluated for oocytes vitrified at GV stage (G2) and for oocytes vitrified at MII stage (G3). Forty-three oocytes out of a total of 45 oocytes vitrified at GV stage (G2) survived the warming process (SR: 95.6 %). Of the 53 GV-oocytes that were in vitro matured before vitrifying (G3), 30 oocytes reached the MII stage and 29 survived devitrification at this stage (SR: 96.7 %). No significant differences were found in the SR after warming between oocytes vitrified at the GV versus MII stages (*P* = 0.810). Therefore, IVMR in G2 was evaluated after the vitrification process, while the IVMR in G3 was evaluated before the vitrification process.

Table 1 shows the results of vitrifying before and after IVM on oocyte viability, IVM and embryo development for non-vitrified oocytes (G1 control group), and for oocytes vitrified before (G2) and after (G3) IVM.

**Table 1:** Effects of vitrifying before and after IVM on oocyte viability, IVM and early embryo development

	G1	G2	G3	*P* -value
IVMR	63.4 % (26/41)	83.7 % (↑) (36/43)	56.6 % (30/53)	0.016
AR	73.1 % (19/26)	80.6 % (29/36)	96.7 % (↑) (29/30)	0.047
DRC	68.4 % (13/19)	82.8 % (24/29)	69.0 % (20/29)	0.397
DRCM	15.8 % (3/19)	20.7 % (6/29)	0.0 % (↓) (0/29)	0.040
DRB	0.0 % (0/19)	3.5 % (1/29)	0.0 % (0/29)	0.432

Group 1 (Control group): 41 GV-oocytes in vitro matured until MII; Group 2 (oocytes vitrified at GV stage): 43 oocytes vitrified at GV stage warmed and in vitro matured until MII and Group 3 (oocytes vitrified at MII stage): 53 GV-oocytes in vitro matured until MII and then vitrified. In vitro oocyte maturation rate (IVMR), oocyte activation rate (AR), development rate to 2-cells embryo at day 2 (DRC), development rate to morula at day 4 (DRCM), development rate to blastocyst at day 5 (DRB). (↑), (↓): The arrow indicates the group that is statistically different from the other two in the ratio value. The direction of the arrow indicates the direction of change in the value of the ratio

The in vitro maturation rate (IVMR) was very similar for oocytes vitrified at MII stage after IVM (G3: 56.6 %) compared to the MII oocytes obtained in the control group (G1: 63.4 %). However, oocytes vitrified before IVM (G3) showed a significantly higher IVMR (83.7 % *p* = 0.016). Although AR was significantly higher for oocytes vitrified at MII stage (G1: AR 73.1 %; G2: AR 80.6 % and G3: AR 96.7 % *p* = 0.047), no significant differences between groups were observed in DRC (G1: 68.4 %, G2: 82.8 % and G3: 69.0 % *p* = 0.397). DRCM was significantly higher for G1 and G2 when compared to G3 (G1: 15.8 %, G2: 20.7 % and G3: 0.0 % *p* = 0.040). Although DRB was only obtained for the oocytes vitrified before IVM, these differences were not statistically significant (G1: 0.0 %, G2: 3.5 % and G3: 0.0 % *p* = 0.432). The only blastocyst obtained in G2 was a poor quality blastocyst at day 5 of development.

### Effects of VS and ionomycin treatment on oocyte viability, IVM, and early embryo development

In order to elucidate the effect of vitrifying solutions (VS) on human oocyte IVM through the intracellular Ca^2+^ oscillation, GV-oocytes were exposed to VS (G4) and ionomycin (G5), which also interact with Ca^2+^ pattern. When this was done, G2 (oocytes vitrified at GV stage) served as a control group.

Table 2 shows the results of the VS and ionomycin treatment on oocyte viability, IVM, and embryo development for G2 (oocytes vitrified at GV stage, control group), G4 (exposed to VS), and G5 (exposed to 10 μM ionomycin).

**Table 2:** Effects of VS and ionomycin treatments on oocyte viability, IVM and early embryo development

	G2	G4	G5	*P* -value
IVMR	83.7 % (36/43)	86.1 % (37/43)	48.8 % (↓) (20/41)	0.000
AR	80.6 % (29/36)	86.5 % (32/37)	55.0 % (↓) (11/20)	0.047
DRC	82.8 % (24/29)	87.5 % (28/32)	90.9 % (10/11)	0.765
DRCM	20.7 % (6/29)	37.5 % (12/32)	0.0 % (↓) (0/11)	0.036
DRB	3.5 % (1/29)	12.5 % (4/32)	0.0 % (0/11)	0.235

Group 2 (control group): 43 oocytes vitrified at GV stage warmed and in vitro matured until MII; Group 4 (exposed to VS): 43 GV-oocytes exposed to vitrifying solutions (VS) without the subsequent immersion in liquid nitrogen (LN2) and Group 5 (Chemical treatment): 41 GV-oocytes exposed to 10 μM ionomycin and in vitro matured until MII. In vitro oocyte maturation rate (IVM-R), oocyte activation rate (AR), development rate to 2-cells embryo at day 2 (DRC), development rate to morula at day 4 (DRCM), development rate to blastocyst at day 5 (DRB). (↑), (↓): The arrow indicates the group that is statistically different from the other two in the ratio value. The direction of the arrow indicates the direction of change in the value of the ratio

The in vitro maturation rate (IVMR) was significantly higher for oocytes exposed to the VS (G4) and for oocytes vitrified at the GV stage (G2 control group) compared to the oocytes exposed to ionomycin in G5 (G2: 83.7 %; G4: 86.1 % and G5: 48.8 % *p* = 0.000). AR was also significantly higher for G2 and G4 when compared to G5 (G2: 80.6 %, G4: 86.5 % and G5: 55.0 % *p* = 0.047). No significant differences between groups were observed in DRC (G2: 82.8 %, G4: 87.5 % and G5: 90.9 % *p* = 0.765). The high DRC obtained in G5 could be due to the ionomycin exposure.

DRCM and DRB were only obtained in G2 and G4. DRCM was significantly higher for oocytes vitrified at the GV stage (G2) and for oocytes exposed to the VS in G4 when compare to the oocytes exposed to the ionomycin in G5 (G2: 20.7 %; G4: 37.5 % and G5: 0.0 % *p* = 0.036). Although DRB was only obtained in G2 and G4, and the blastocyst percentage was higher for G4, no significant differences were found (G2: 3.5 %; G4: 12.5 % and G5: 0.0 %; *p* =0.235). This lack of difference may result from the low number of obtained blastocysts.

**Fig. 2:**
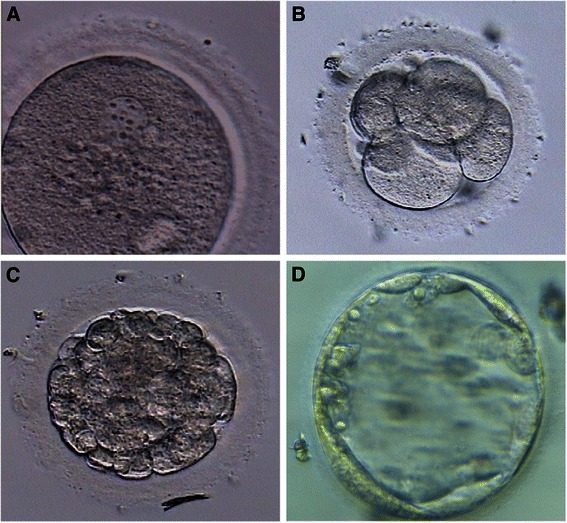
Oocyte and embryo development. Parthenogenetic embryo development of GV-oocytes exposed to vitrification solutions without the subsequent immersion in LN_2_ and in vitro matured until MII (G4). Activated oocyte showing an elongated pronucleus, which had not expelled the second polar body (**a**). Parthenogenetic embryo at day 2 (**b**), at day 4 (morura stage) (**c**), and at day 5 (blastocyst stage) (**d**) of development

In addition, the four blastocysts obtained in G4 were also poor quality blastocysts at day 5 of development (Fig. 2).

## Discussion

This is the first study with human GV-oocytes to evaluate the effects of vitrifying before and after IVM. It is also the first to assess exposure to the VS and ionomycin on oocyte viability and embryo development by parthenogenetic activation. Our findings demonstrate that the osmotic effect of the cryoprotectants included in the VS resulted in a higher IVM potential, as well as improved embryo cleavage and development. This clarifies the effect of vitrification solutions in IVM potential and also establishes the best oocyte stage for performing the vitrification and IVM procedures. This provides an additional tool in clinical practice to increase the pool of mature oocytes in patients with a high risk of developing premature ovarian failure, or when controlled ovarian stimulation is contraindicated, as observed in some oncological patients.

The knowledge of the effects of vitrification protocols on oocyte viability, as well optimizing the cryopreservation of immature oocytes before or after IVM, is essential. This is particularly important for pre-pubertal girls with hematologic malignant diseases in which the auto-transplantation of ovarian tissue (OT) is contraindicated.

For fertility preservation purposes, oocytes (immature or mature) should be cryopreserved at some point. Protocols should also be developed which allow the storage of a large number of oocytes in an appropriate stage of maturation for future uses.

It has been reported that a significant number of immature oocytes can be collected from excised OT regardless of the menstrual cycle phases or the age of the patients, even for pre-pubertal girls [30] and premenopausal patients [31].

There is a limited quantity of human OT donated for research, making GV-oocytes from unstimulated ovaries difficult to obtain. Immature oocytes from stimulated and unstimulated cycles show similar behavior resulting in chromosomally competent blastocysts [3]. Therefore, GV-oocytes unsuitable for ICSI cycles were used in this study.

The oocyte is unique in that the maternal DNA is suspended in the cytoplasm on the meiotic spindle and not within the protective confines of the nuclear membrane, as it typically is during subsequent pre-implantation development. Damage to the DNA and/or microtubules could explain the limited success of oocyte cryopreservation. However, a further consideration is that the oocyte is arrested in a state primed for activation, and changes in its environment could cause parthenogenetic activation [25]. Many authors have proposed that the immature oocyte might prove less vulnerable to cryopreservation-induced injuries than the mature MII oocyte due to the absence of the temperature- and chemically-sensitive meiotic spindle in GV-oocytes [30, 32].

There are few studies comparing the vitrification process before and after IVM. It has been demonstrated that vitrified immature oocytes can undergo post-warming in vitro maturation and fertilization, and also produce embryos able to undergo vitrification and warming [33] without generating chromosomal abnormalities [3]. Related to the SR of vitrified oocytes before and after IVM, similar SR have been reported for MII (86.9 %) and GV-oocytes (84 %) [34]. Survival rates are significantly higher for GV-oocytes (90.1 %) than MII (64.7 %), regardless of the vitrification protocol used [32].

In this study, the SR after warming obtained for oocytes vitrified at the GV stage (G2: 95.5 %) was very similar to those obtained for oocytes vitrified at the MII stage (G3: 96.6 %). These results corroborate previous studies indicating that the SR is not related to the time of vitrification (before or after in vitro maturation). Therefore, immature oocytes at the GV stage seem to be more suitable for oocyte storage in the case of oncology patients who urgently need to preserve their fertility, especially when controlled ovarian stimulation is contraindicated.

The next question that arises is whether IVM potential can be lost due to the cryopreservation process in oocytes vitrified at GV. DMSO has been shown to cause a transient intracellular Ca^2+^ rise in various cell lines [21], and high concentrations of ethylene glycol (EG, 10–40 %) also increase intracellular Ca^2+^ in mouse oocytes [22]. Therefore, a potential problem with oocyte freezing is the induction of a primary activation event.

The DMSO appeared to induce a larger and more sustained increase in Ca^2+^ compared with EG, which may be attributable to the higher permeability of the oocyte to DMSO [35]. The permeability of the oocyte to cryoprotectants and their ability to replace intracellular water is another well-documented finding [36, 37]. The lipophilic properties of DMSO and EG would be expected to have a non-specific effect on the plasma membrane and other internal membranes, such as the endoplasmic reticulum (ER), which would lead to either Ca^2+^ influx and/or Ca^2+^ release from internal stores. However, the osmotic contraction induced by cryoprotectants might also contribute to the increase in intracellular Ca^2+^.

Ca^2+^ oscillations are essential for embryonic development. The ability to generate Ca^2+^ oscillations develops in mammalian oocytes during meiotic maturation (from GV to MII) and depends more on cytoplasmic changes than the progression of meiosis [38].

Oocyte cryopreservation and in vitro culture affect Ca^2+^ signaling during human fertilization, and in vivo matured human oocytes exhibit a distinct pattern of Ca^2+^ oscillations compared to IVM and in vitro aged oocytes [39]. Moreover, Ca^2+^ concentration in vitrifying medium affects the developmental potential of in vitro matured ovine oocytes [40].

Andjuk et al. [38] indicated that several cytoplasmic changes are required to generate Ca^2+^ oscillations, including reorganization of ER (the main stockpile of Ca^2+^ in the oocyte) and an increase in the concentration of Ca^2+^ ions stored in ER.

The osmotic effect of the cryoprotectants included in the VS resulted in a higher IVM potential and early embryo development to blastocyst stage for osmotic treatment (G4) and for oocytes vitrified at the GV stage (G2). Oocytes subjected only to an osmotic shock produced the highest rates of IVMR and early embryo development, even higher than those obtained for oocytes vitrified before IVM, demonstrating that oocyte activation is mainly due to the effect of VS.

It is well know that ER, the major oocyte Ca^2+^ store, undergoes a profound reorganization during oocyte maturation which affects Ca^2+^ oscillations [41]. Furthermore, the exposure of mammalian oocytes to VS, using DMSO as a cryoprotectant, causes an increase in the concentration of intracellular Ca^2+^ [25, 26]. Therefore, it is reasonable to assume that the DMSO included in the VS in this study increased intracellular Ca^2+^ concentrations, which stimulated the IVM in those groups where GV-oocytes were vitrified or exposed to VS prior to the IVM procedure.

The IVMR for GV subjected to the osmotic treatment and for oocytes vitrified at the GV stage (G4: 86 %; G2: 83.7 %) were higher than those previously achieved by other groups such as Isachenco et al., (72 %) [42] and Cao et al., (70.4 %) [43].

The IVMR was similar for oocytes vitrified at the MII stage (G3: 56.6 %) and for non-vitrified MII oocytes in the control group (G1: 63.4 %), but it was lower than those achieved by Söderström-Anttila et al.*,* (62.6 %) [44] and by Roesner et al., (64.9 %) [45]. This data is not comparable, however, because those authors used specific IVM culture media, whereas in this study only conventional IVF medium was used. Conventional IVF medium was also used for IVM in G4 and G2, but in these cases the oocytes were exposed to the VS before the IVM and IVMR were obtained. These results suggest that the Ca^2+^ released from the ER would be more important for oocyte maturation than specific IVM culture media.

These results also demonstrate that the thermal shock produced by the immersion in LN_2_ has an unfavorable effect on early embryonic development. Unfortunately, the thermal shock cannot be evaluated separately from the osmotic shock in the vitrifying procedures.

The high DRC obtained in oocytes exposed to ionomycin (G5, chemical treatment) could be because ionomycin enters the membrane of intracellular organelles and allows the release of Ca^2+^ ions. These discrepancies have been previously explained as a different accessibility of ER to the ionophore in GV and MII oocytes [28]. That work assumed that the ionophores might more effectively insert into the membranes of clustered ER in the MII stage than into the membranes of more diffused ER structures in GV oocytes. This is why ionomycin does not promote IVM at the GV stage.

The poor quality of the blastocysts obtained in this study could be due to the fact that all of them were obtained from IVM and parthenogenetically activated GV-oocytes without sperm contribution to the fertilization process, as the blastocyst obtained from mature MII vitrified oocytes allow good quality blastocysts to be obtained at day 5 after ICSI. However, this is the only ethical and legal procedure in our country to assess oocyte competence and viability for research purposes.

The Ca^2+^ oscillations induced by the high concentration of cryoprotectants in the VS (4 or 5 times higher than those used for conventional freezing) could be responsible for both the higher rates of IVM and the better subsequent embryonic development.

Comparing the results of G1 and G2 shows that IVM and subsequent embryo viability are enhanced by the vitrification process. May be the oocyte aging caused by in vitro culture from GV to MII stage determines this result. May be the oocyte in vitro aging due to in vitro culture from GV to MII stage determines this result. In any case, these data suggest that the results are better after GV-oocyte vitrification. The comparison of G2 and G3 confirms that the vitrification process improves IVM. On the other hand, when we vitrified oocytes in the MII stage (G3), a high AR and good DRC were achieved. These rates were also similar to those found in the control group, although no blastocysts were obtained. These findings support those in IVF-ICSI cycles in which only GV-oocytes were obtained. In these cases, we performed ICSI in the MII oocytes after IVM and, despite obtaining good quality embryos, no pregnancies were achieved possibly due to the fact that the embryos acquired in these IVF-ICSI cycles and in G3 (oocytes vitrified at MII stage) did not reach the blastocyst stage.

This study has some limitations. First, the GV-oocytes from stimulated cycles do not accurately represent the unstimulated cycles in cancer patients. However, GV-oocytes discarded from ICSI cycles are an ethical and legal biological material that can be used to study cryopreservation and IVM protocols for clinical applications. Second, during vitrification procedures, the thermal shock cannot be evaluated separately from the osmotic shock. As the fertilization of human oocytes for research purposes is not allowed, it must be taken into account that the developmental capacity of a cell cluster is not necessarily equivalent to the developmental capacity after “true” fertilization.

It is evident that the clinically optimal conditions for obtaining good quality embryos include the fertilization of fresh or vitrified mature oocytes. However, the results obtained in this study from PI are encouraging, especially for those patients with a high risk of premature ovarian failure or with a very low ovarian response, in which it is necessary to recover the greatest possible number of oocytes. Thus, the PI oocytes that are discarded after the ovarian puncture could be vitrified because, as we observed in this study, vitrification solutions improve the GV IVM process after thawing.

## Conclusion

The findings from this study indicate that oocyte quality is not affected by their maturation stage at the vitrifying time. Results also indicate that VS improves IVM of GV-oocytes and early embryonic development. Vitrifying GV-oocytes for subsequent IVM can permit the storage of a large number of oocytes in an appropriate stage of maturation for future use. Cryopreservation of ovarian cortex for subsequent autologous orthotransplantation is the most widely used technique to preserve fertility in oncologic patients, and it is the only option in cases of hormone-dependent diseases and for pediatric patients who have no mature oocytes to be cryopreserved. However, the GV-oocytes present in the oophorectomy specimen are lost before the cryopreservation process. Future studies using this methodology could be useful for increasing the pool of oocytes and improving FP options.

## References

[1] Hong SW, Chung HM, Lim JM, Ko JJ, Yoon TK, Yee B, Cha KY (1999). Improved human oocyte development after vitrification: a comparison of thawing methods. Fertil Steril.

[2] Kuleshova L, Gianaroli L, Magli C, Ferraretti A, Trounson A (1999). Birth following vitrification of a small number of human ocoytes: case report. Hum Reprod.

[3] Chung HM, Hong SW, Lim JM, Lee SH, Cha WT, Ko JJ, Han SY, Choi DH, Cha KY (2000). In vitro blastocyst formation of human oocytes obtained from unstimulated and stimulated cycles after vitrification at various maturational stages. Fertil Steril.

[4] Yoon TK, Kim TJ, Park SE, Hong SW, Ko JJ, Chung HM, Cha KY (2003). Live births after vitrification of oocytes in a stimulated in vitro fertilization-embryo transfer program. Fertil Steril.

[5] Yoon TK, Lee DR, Cha SK, Chung HM, Lee WS, Cha KY (2007). Survival rate of human oocytes and pregnancy outcome after vitrification using slush nitrogen in assisted reproductive technologies. Fertil Steril.

[6] Kuwayama M, Vajta G, Kato O, Leibo SP (2005). Highly efficient vitrification method for cryopreservation of human oocytes. Reprod Biomed Online.

[7] Kyono K, Fuchinoue K, Yagi A, Nakajo Y, Yamashita A, Kumagai S (2005). Successful pregnancy and delivery after transfer of a single balstocyst derived from a vitrified mature human oocyte. Fertil Steril.

[8] Lucena E, Bernal DP, Lucena C, Rojas A, Moran A, Lucena A (2006). Successful ongoing pregnancies after vitrification of oocytes. Fertil Steril.

[9] Vajta G, Nagy ZP (2006). Are programmable freezers still needed in the embryo laboratory? Review on vitrification. Reprod Biomed Online.

[10] Camus A, Clairaz P, Ersham A, Kappel AL, Savic G, Staub C (2006). The comparison of the process of five different vitrification devices. Gynecol Obstet Fertil.

[11] Selman H, Angelini A, Barnocchi N, Brusco GF, Pacchiarotti A, Aragona C (2006). Ongoing pregnancies after vitrification of human oocytes using a combined solution of ethylene glycol and dimethyl sulfoxide. Fertil Steril.

[12] Antinori M, Licata E, Dani G, Cerusico F, Versaci C, Antinori S (2007). Cryotop vitrification of human oocytes results in high survival rate and healthy deliveries. Reprod Biomed Online.

[13] Committee IP, Kim SS, Donnez J, Barri P, Pellicer A, Patrizio P, Rosenwaks Z, Nagy P, Falcone T, Andersen C (2012). Recommendations for fertility preservation in patients with lymphoma, leukemia, and breast cancer. J Assist Reprod Genet.

[14] Coticchio G, Dal-Canto M, Guglielmo MC, Mignini-Renzini M, Fadini R (2012). Human oocyte maturation in vitro. Int J Dev Biol.

[15] Eppig JJ (1996). Coordination of nuclear and cytoplasmic oocyte maturation in eutherian mammals. Reprod Fertil Dev.

[16] Carafoli E (2002). Calcium signaling: a tale for all seasons. Proc Natl Acad Sci U S A.

[17] Berridge MJ, Bootman MD, Roderick HL (2003). Calcium signalling: dynamics, homeostasis and remodelling. Nat Rev Mol Cell Biol.

[18] Homa S (1995). Calcium and meiotic maturation of the mammalian oocyte. Mol Reprod Dev.

[19] Carroll J, Swann K, Whittingham D, Whitaker M (1994). Spatiotemporal dynamics of intracellular [Ca2+]i oscillations during the growth and meiotic maturation of mouse oocytes. Development.

[20] Jones KT, Carroll J, Whittingham DG (1995). Ionomycin, thapsigargin, ryanodine, and sperm induced Ca2+ release increase during meiotic maturation of mouse oocytes. J Biol Chem.

[21] Morley P, Whitfield JF (1993). The differentiation inducer, dimethyl sulfoxide, transiently increases the intracellular calcium ion concentration in various cell types. J Cell Physiol.

[22] Takahashi T, Igarashi H, Doshida M, Takahashi K, Nakahara K, Tezuka N, Kurachi H (2004). Lowering intracellular and extracellular calcium contents prevents cytotoxic effects of ethylene glycolbased vitrification solution in unfertilized mouse oocytes. Mol Reprod Dev.

[23] Kline D, Kline JT (1992). Repetitive calcium transients and the role of calcium in exocytosis and cell cycle activation in the mouse egg. Dev Biol.

[24] Tahara M, Tasaka K, Masumoto N, Mammoto A, Ikebuchi Y, Miyake A (1996). Dynamics of cortical granule exocytosis at fertilization in living mouse eggs. Am J Physiol.

[25] Larman MG, Sheehan CB, Gardner DK (2006). Calcium-free vitrification reduces cryoprotectant-induced zona pellucida hardening and increases fertilization rates in mouse oocytes. Reproduction.

[26] Kohaya N, Fujiwara K, Ito J, Kashiwazaki N (2011). High developmental rates of mouse oocytes cryopreserved by an optimized vitrification protocol: the effects of cryoprotectants, calcium and cumulus cells. J Reprod Dev.

[27] Debon A, Molina I, Cabrera S, Pellicer A (2012). Mathematical methodology to obtain and compare different embryo scores. Math Comput Model.

[28] Paffoni A, Brebini T, Somigliana E, Restelli L, Gandolfi F, Ragni G (2007). In vitro development of human oocytes after parthenogenetic activation or intracytoplasmic sperm injection. Fertil Steril.

[29] Gardner DK, Schoolcraft WB, Jansen R, Mortimer D (1999). In vitro culture of human blastocyst. Towards reproductive certainty: infertility and genetics beyond 1999.

[30] Fasano G, Demeestere I, Englert Y (2012). In-vitro maturation of human oocytes: before or after vitrification?. J Assist Reprod Genet.

[31] Imesch P, Scheiner D, Xie M, Fink D, Macas E, Dubey R, Imthurn B (2013). Developmental potential of human oocytes matured in vitro followed by vitrification and activation. J Ovarian Res.

[32] Zhang ZG, Liu Y, Xing Q, Zhou P, Cao Y (2011). Cryopreservation of human failed-matured oocytes followed by in vitro maturation: vitrification is superior to the slow freezing method. Reprod Biol Endocrinol.

[33] Asimakopoulos B, Kotanidis L, Nikolettos N (2011). In vitro maturation and fertilization of vitrified immature human oocytes, subsequent vitrification of produced embryos, and embryo transfer after thawing. Fertil Steril.

[34] Fasano G, Moffa F, Dechène J, Englert Y, Demeestere I (2011). Vitrification of in vitro matured oocytes collected from antral follicles at the time of ovarian tissue cryopreservation. Reprod Biol Endocrinol.

[35] Paynter S (2005). A rational approach to oocyte cryopreservation. Reprod Biomed Online.

[36] Pfaff RT, Liu J, Gao D, Peter AT, Li TK, Crister JK (1998). Water and DMSO membrane permeability characteristics of in vivo- and in vitro-derived and cultured murine oocytes and embryos. Mol Hum Reprod.

[37] Pedro PB, Yokoyama E, Zhu SE, Yoshida N, Valdez DM, Tanaka M, Edashige K, Kasai M (2005). Permeability of mouse oocytes and embryos at various developmental stages to five cryoprotectants. J Reprod Dev.

[38] Ajduk A, Małagocki A, Maleszewski M (2008). Cytoplasmic maturation of mammalian oocytes: development of a mechanism responsible for sperm-induced Ca2+ oscillations. Reprod Biol.

[39] Nikiforaki D, Vanden Meerschaut F, Qian C, De Croo I, Lu Y, Deroo T, Van den Abbeel E, Heindryckx B, De Sutter P (2014). Oocyte cryopreservation and in vitro culture affect calcium signalling during human fertilization. Hum Reprod.

[40] Succu S, Berlinguer F, Leoni GG, Bebbere D, Satta V, Marco-Jimenez F, Pasciu V, Naitana S (2011). Calcium concentration in vitrification medium affects the developmental competence of in vitro matured ovine oocytes. Theriogenology.

[41] Kline D (2000). Attributes and dynamics of the endoplasmic reticulum in mammalian eggs. Curr Top Dev Biol.

[42] Isachenko V, Montag M, Isachenko E, Dessole S, Nawroth F, Van der Ven H (2006). Aseptic vitrification of human germinal vesicle oocytes using dimethyl sulfoxide as a cryoprotectant. Fertil Steril.

[43] Cao Y, Xing Q, Zhang ZG, Wei ZL, Zhou P, Cong L (2009). Cryopreservation of immature and in-vitro matured human oocytes by vitrification. Reprod Biomed Online.

[44] Soderstrom-Anttila V, Makinen S, Tuuri T, Suikkari AM (2005). Favorable pregnancy results with insemination of in vitro matured oocytes from unstimulated patients. Hum Reprod.

[45] Roesner S, Von Wolff M, Eberhardt I, Beuter-Winkler P, Toth B, Strowitzki T (2012). In vitro maturation: a five-year experience. Acta Obstet Gynecol Scand.

